# HIV-Associated Cryptococcal Immune Reconstitution Inflammatory Syndrome Is Associated with Aberrant T Cell Function and Increased Cytokine Responses

**DOI:** 10.3390/jof5020042

**Published:** 2019-05-23

**Authors:** David B. Meya, Samuel Okurut, Godfrey Zziwa, Stephen Cose, David R. Boulware, Edward N. Janoff

**Affiliations:** 1 Infectious Diseases Institute, Makerere University, Kampala P.O. Box 22418, Uganda; okuruts@gmail.com; 2 Department of Medicine, Center for Infectious Diseases and Microbiology Translational Research, University of Minnesota, Minneapolis, MN 55455, USA; boulw001@umn.edu; 3 School of Medicine, College of Health Sciences, Makerere University, Kampala P.O. Box 7072, Uganda; 4 Research Department, Makerere University Walter Reed Project, Plot 42, Nakasero Road, Kampala P.O. Box 1624, Uganda; gzziwa@muwrp.org; 5 Clinical Research Department, London School of Hygiene & Tropical Medicine, Keppel Street, London WC1E 7HT, UK; stephen.cose@mrcuganda.org; 6 MRC/UVRI and LSHTM Uganda Research Unit, Plot 51–59 Nakiwogo Road, Entebbe P.O.Box 49, Uganda; 7 Mucosal and Vaccine Research Program Colorado (MAVRC), University of Colorado, Denver, Aurora, CO 80045, USA; Edward.Janoff@ucdenver.edu

**Keywords:** cryptococcal meningitis, *Cryptococcus*, HIV, CD4 T cells, CD8 T cells, adaptive immune response, IRIS

## Abstract

Cryptococcal meningitis remains a significant opportunistic infection among HIV-infected patients, contributing 15–20% of HIV-related mortality. A complication of initiating antiretroviral therapy (ART) following opportunistic infection is immune reconstitution inflammatory syndrome (IRIS). IRIS afflicts 10–30% of HIV-infected patients with cryptococcal meningitis (CM), but its immunopathogenesis is poorly understood. We compared circulating T cell memory subsets and cytokine responses among 17 HIV-infected Ugandans with CM: 11 with and 6 without CM-IRIS. At meningitis diagnosis, stimulation with cryptococcal capsule component, glucuronoxylomannan (GXM) elicited consistently lower frequencies of CD4^+^ and CD8^+^ T cell memory subsets expressing intracellular cytokines (IL-2, IFN-γ, and IL-17) among subjects who subsequently developed CM-IRIS. After ART initiation, T cells evolved to show a decreased CD8^+^ central memory phenotype. At the onset of CM-IRIS, stimulation more frequently generated polyfunctional IL-2^+^/IL-17^+^ CD4^+^ T cells in patients with CM-IRIS. Moreover, CD8^+^ central and effector memory T cells from CM-IRIS subjects also demonstrated more robust IL-2 responses to antigenic stimulation vs. controls. Thus, ART during CM elicits distinct differences in T cell cytokine production in response to cryptococcal antigens both prior to and during the development of IRIS, suggesting an immunologic foundation for the development of this morbid complication of CM infection.

## 1. Introduction

Cryptococcal meningitis causes 15–20% of AIDS-related mortality worldwide [1]. In sub-Saharan African countries with high HIV prevalence (>5%), *Cryptococcus* is the most common cause of meningitis in adults, accounting for 26% of cases in Malawi, 45% in Zimbabwe, 30% in South Africa [2,3,4,5], and 60% in Uganda [3,5,6]. In 2014, an estimated 162,500 cases of cryptococcal meningitis (CM) occurred in sub-Saharan Africa resulting in more than 90,000 deaths [1]. Despite improved immune function in antiretroviral therapy (ART)-treated HIV-infected patients in low and middle-income countries, a significant proportion of patients with a new HIV diagnosis still present with advanced disease (CD4^+^ T cells < 200/μL) and are at risk for opportunistic infections (OI) such as cryptococcal meningitis [7]. 

ART suppresses HIV replication and CD4^+^ T cell loss by apoptosis allowing immune reconstitution to occur. However, in addition to its benefits, immune reconstitution with ART can also be detrimental. A proportion of patients treated with ART experience a constellation of symptoms and signs in which sub-clinical or pre-existing infections trigger an exaggerated inflammatory response that leads to clinical deterioration, presenting as immune reconstitution inflammatory syndrome (IRIS) [8]. IRIS can present as unmasking or paradoxical phenomena. In unmasking IRIS, subclinical infections become overtly symptomatic with a first episode of the OI after ART initiation. Conversely, in paradoxical IRIS, there is usually evidence of initial microbiological and clinical response to treatment of the OI pre-ART, which evolves into the recrudescence of symptoms following ART without microbiological evidence of the associated OI following ART initiation.

Depending on the site and activity, IRIS can present with a range of symptoms from minor to severe inflammation resulting in organ failure, hospitalization, or death [9,10,11,12]. Whether the current shift to a ‘Test & Treat’ ART strategy with limited screening and treatment of OIs prior to ART will increase the incidence of unmasking IRIS remains to be seen. Reversal of CD4^+^ T cell lymphopenia and increased T cell activation have been associated with the development of IRIS [13]. Studies in vitro have demonstrated that attenuated *Cryptococcus*-specific IFN-γ responses prior to starting ART are associated with cryptococcal meningitis-IRIS when patients who developed CM-IRIS were compared to HIV-infected controls who did not develop IRIS [14]. However, the ontogeny of antigen-specific T cell responses prior to and during cryptococcal IRIS are not well defined.

We have previously demonstrated that lower levels of inflammation, demonstrated by decreased numbers of CSF leukocytes and levels of CSF protein, IFN-γ, IL-6, IL-8, and TNF-α were predictors for developing cryptococcal IRIS [15]. It is imperative that the mechanisms underlying cryptococcal IRIS are understood in order to prevent or optimize interventions against the deleterious effects of cryptococcal IRIS. We hypothesized that prior to ART initiation, T cell phenotype and function would distinguish patients who subsequently did and did not later develop paradoxical cryptococcal IRIS. We, therefore, investigated the quantitative and functional reconstitution of CD4^+^, CD8^+^ T cells, characterizing the association of T cell responses with the development or absence of cryptococcal IRIS in HIV-infected patients receiving ART after cryptococcal meningitis treatment in order to understand the contribution of these components to the immunopathogenesis of cryptococcal IRIS.

## 2. Materials and Methods

### 2.1. Study Subjects and Procedures

As previously described [16], we sequentially screened participants presenting with suspected meningitis at Mulago National Referral Hospital in Kampala, Uganda. We enrolled adults with a first episode of CM diagnosed by cerebrospinal fluid (CSF) cryptococcal antigen or positive *Cryptococcus neoformans* culture [17]. Participants received amphotericin B (0.7–1 mg/kg/day) for 2 weeks with oral fluconazole (800 mg/day) which was continued for ~5 weeks, then later decreased to 400 mg/day for 8 weeks and 200 mg/day thereafter [18]. ART (zidovudine, lamivudine, and efavirenz) was started within 6 weeks of CM diagnosis [19].

We collected blood from subjects longitudinally with the isolation of peripheral blood mononuclear cells (PBMCs) by density centrifugation gradient (Ficoll 1077, Sigma) followed by cryopreservation in RPMI-1640 with fetal bovine serum (20%), dimethyl sulphoxide (10%), and penicillin/streptomycin, 1%) in liquid nitrogen.

A diagnosis of definite/probable/possible CM-IRIS was made according to the published consensus case definition [8], with external adjudication by a three-physician panel whose members were not part of the clinical team. Grading was classified as definite, probable, or possible IRIS based on the available clinical and CSF information. Institutional review board approvals were obtained from the School of Medicine Ethics review committee at Makerere University (REF 2009–022) and the University of Minnesota (0810M49622), and written informed consent was obtained.

### 2.2. PBMC Stimulation and Surface Flow Cytometric Staining

Cryopreserved PBMCs at initial CM diagnosis, during CM-IRIS and from control subjects without CM-IRIS matched for ART duration were thawed and stimulated in vitro. PBMCs were rapidly thawed and diluted in complete media (RPMI-1640 with 10% FBS, 2% HEPES, 2% l-Glutamine, 1% Pen/Strep) and 1 × 10^6^ cells were added to each of three wells. Glucuronoxylomannan (GXM) was added to the test well with co-stimulatory anti-CD28 and anti-49d monoclonal antibodies to enhance the detection of cytokine-secreting T cells. Staphylococcal enterotoxin B (SEB; 10 ng/mL) (Invivogen, France), a non-specific polyclonal activator, was used as the positive control and phosphate buffered saline (PBS) (100 μL/well) (Sigma-Aldrich, USA) as the negative control. After 2 hours of incubation in 5% carbon dioxide at 37 °C, Brefeldin A (100 μL/mL) (BD, Golgi Stop, catalog # 554724) was added to each well (to inhibit intracellular transport, allowing for the accumulation of cytokines in the Golgi complex) and cells were incubated for another 4 hours then refrigerated in the dark overnight at 4 °C.

**Figure 1:**
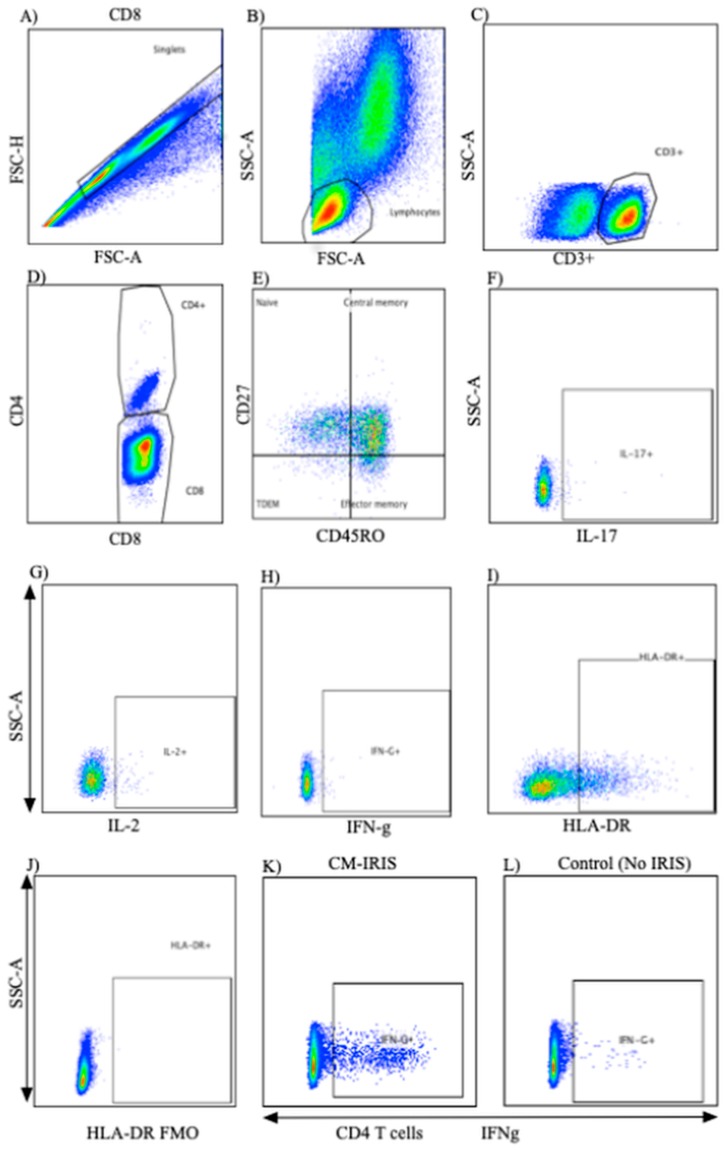
Flow cytometry gating strategy for T cells. Multiparameter flow cytometry was used to identify the frequency, phenotype, and post-stimulation cytokine expression of CD4^+^ and CD8^+^ T cells within total peripheral blood mononuclear cells of patients with and without CM-IRIS at baseline and at the CM-IRIS event. Representative staining shows the analytic gating strategy: (**A**) FSC-H/FSC-A showing the singlet gate; (**B**) FSC/SSC for lymphocytes selected from singlet gate; (**C**) T cells expressing CD3 were selected; (**D**) CD3^+^ cells expressing CD4 and CD8 were then identified; (**E**) gating by differential expression of CD27 and CD45RO identified naïve and memory T cells subsets with naive T cells as (CD27^+^CD45RO^−^); central memory as (CD27^+^CD45RO^+^), effector memory as (CD27^−^CD45RO^+^), and terminally differentiated effector memory as (CD27^−^CD45RO^−^); (**F**) CD4^+^ expression of IL-17 (unstimulated); (**G**) representative example of IL-2 expression by CD4^+^ T cells (unstimulated); (**H**) representative example of IFN-γ expression by CD4^+^ T cells (unstimulated); (**I**) representative example of HLA-DR expression by CD4^+^ T cells and; (**J**) HLA-DR Fluorescence minus one gating; (**K**) representative example of IFN-γ expression by CD4^+^ T cells in a subject with CM-IRIS and; (**L**) IFN-γ expression by CD4^+^ T cells in a control subject without CM-IRIS.

We assessed in vitro cytokine responses to cryptococcal GXM in circulating T cells at CM diagnosis, at the time of CM-IRIS, or a matched ART time point for CM controls without IRIS. We stained cells with commercial monoclonal antibodies reactive with CD45RO^PerCPCy5.5^ (clone UCHL1, BD Biosciences), CD27^APC-H7^ (clone MT271, BD Biosciences), CD3^V500^ (clone UCHT1, BD Biosciences), and CD4^V450^ (clone RPA-T4, BD Horizon). CD3^+^CD4^−^ cells were considered as CD8^+^ T cells. We assessed T cell activation using the proportion of T cells expressing HLA-DR^PECy7^ (clone LN3, BD Biosciences). We prepared fluorescence minus one (FMO) controls on blood samples to set gates for CD27 and CD45RO. Following the selection of single cells based on FSC height vs. FSC area, differential gating of lymphocytes was based on size and granularity (Figure 1). T cell phenotype, activation state, and percentage of T cell subsets were determined by 8-color flow cytometry using a FACSCanto II (BD Biosciences).

### 2.3. Intracellular Cytokine Staining

We determined T cell cytokine responses by intracellular cytokine staining. Following 6 hours of stimulation at 37 °C and overnight incubation at 4 °C, cells were washed, fixed and permeabilized with successive washes in FACS Permeabilizing Solution (BD Biosciences) and stained with intracellular monoclonal antibodies reactive with IFN-γ^PE^ (clone 4S.B3, Biolegend), and IL-17^Alexa647^ (clone SPCL 1362, BD Biosciences) and IL-2^FITC^ (clone 5433.111, BD Biosciences). One million events were acquired the following day using an 8-color FACS Canto II (BD Biosciences), and data were analyzed using FlowJo version 10.0.5 (TreeStar, USA).

### 2.4. Statistical Analysis

Data were analyzed using GraphPad Prism, version 6.0b (GraphPad Software Inc., CA, USA) and Spice, version 5.35 (NIAID, NIH, Bethesda, MD, USA). We compared paired samples at meningitis diagnosis and at CM-IRIS event using the non-parametric Wilcoxon signed-rank test. We compared cell phenotype and activation variables between CM-IRIS and time-matched controls using the non-parametric Mann-Whitney rank sum test. Statistical significance was defined as a *p*-value ≤ 0.05. We compared cytokine expression profiles by permutation analysis to determine combinations of cytokine expression following background subtraction, using a 10,000-iteration Monte Carlo simulation model described in detail elsewhere [20].

## 3. Results

Among 11 HIV-1-infected adults who developed CM-IRIS cases, cryopreserved PBMCs were available from 10 at CM diagnosis and 11 at the time of CM-IRIS event. Among the six HIV-1-infected control subjects with CM but no IRIS, PBMC were available from five at the time of the CM diagnosis and all at a subsequent visit time-matched to the timing of CM-IRIS in the other group (67 vs. 78 days).

Age, baseline CD4 and CD8 T cell counts, cryptococcal antigen (CrAg) titer, HIV viral load, CSF protein, and white blood cells were similar among subjects with CM-IRIS vs. controls (Table 1). At meningitis diagnosis, subjects who subsequently developed CM-IRIS showed higher but non-significant quantitative CSF *Cryptococcus* colony forming units (CFU) on culture, median 213,796 (IQR: 91,201–288,403) CFU/mL compared to control subjects who did not develop IRIS median 9332 (IQR: 281–181,970) CFU/mL.

### 3.1. T Cell Phenotype and Activation at CM Diagnosis vs. Controls

**Figure 2:**
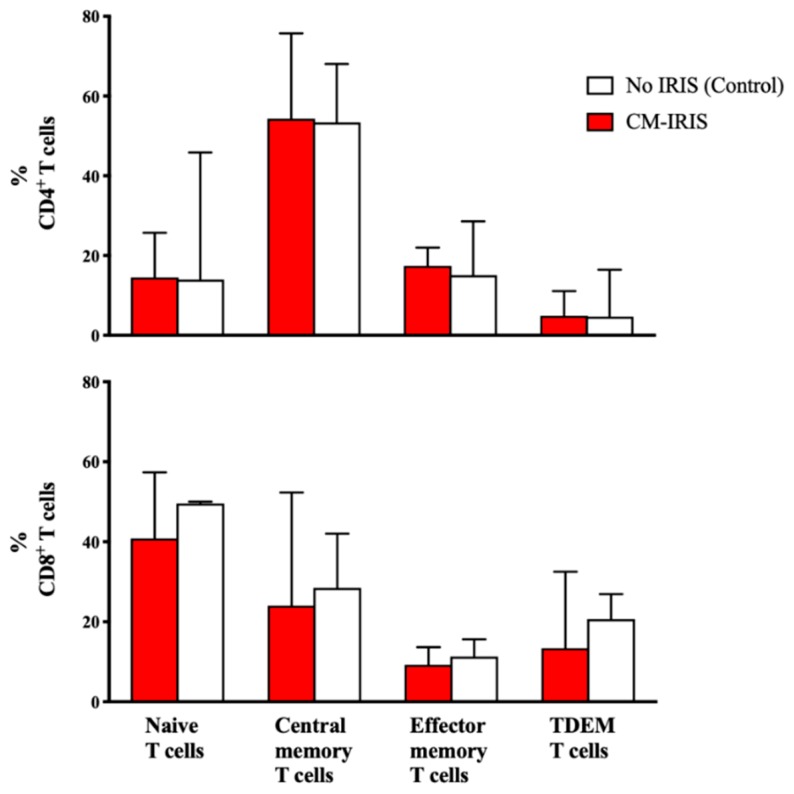
Frequencies of CD4^+^ and CD8^+^ memory T cell subsets at the time of initial cryptococcal meningitis diagnosis among ART-naïve subjects who later developed CM-IRIS vs. controls without IRIS. CD4^+^ Central memory and CD8^+^ naïve T cells predominated without significant differences between groups. Bars represent median values and error bars show interquartile ranges. Abbreviations: TDEM- terminally differentiated effector memory. White bars represent CM diagnosis, red bars represent CM-IRIS.

At CM diagnosis, circulating CD4^+^ T cell numbers were similarly very low in both groups. The frequencies of total CD8^+^ T cells were comparable. Central memory CD4^+^ T cells (CD27^+^CD45RO^+^) and naive CD8^+^ T cells (CD27^+^CD45RO^−^) were the predominant T cell subsets (Figure 2) without significant differences in T cell subsets between groups. Similarly, baseline activation of CD4^+^ T cells expressing HLA-DR was very high at baseline but comparable among subjects with future CM-IRIS vs. controls, 81% (IQR: 66, 90) vs. 72% (IQR: 46, 80), (*p* = 0.196), respectively. Further, CD8 T cell activation, also high, did not differ, with frequencies of 91% IQR: 85, 95) vs. 94% (IQR: 59, 98), (*p* = 0.853) in the two groups, respectively.

### 3.2. T Cell Cytokine Responses at Baseline

At baseline, the frequencies of CD4^+^ and CD8^+^ memory subset T cells expressing intracellular cytokines (IL-2, IFN-γ, and IL-17) after GXM stimulation were consistently lower among subjects who later developed CM-IRIS (Table 2) for each subset and each intracellular cytokine. Unstimulated and SEB stimulation did not demonstrate significant differences in responses. 

### 3.3. Cytokine Responses at CM-IRIS vs. Controls

**Figure 3:**
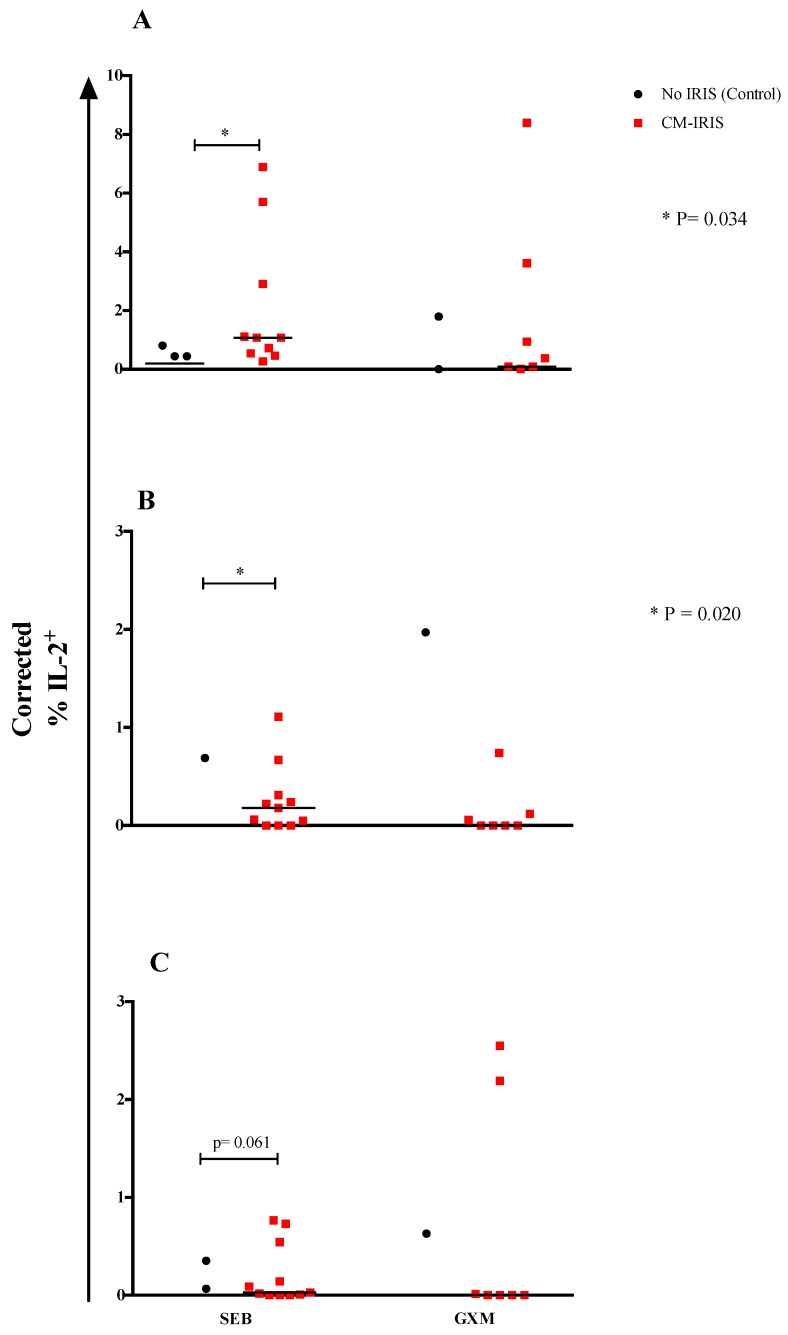
Corrected IL-2 responses by total CD8^+^ T cells (**A**), naïve CD8^+^ T cells (**B**) and CD8^+^ TDEM T cells (**C**) were calculated by subtracting the value for unstimulated samples from the value for mitogen- or IFN-γ-stimulated samples of subjects who developed CM-IRIS compared to controls at baseline. Negative corrected values were reported as zero. Individual responses are shown with the median as a black horizontal line. *p*-values were determined using the Mann Whitney *U* test.

Upon correcting for the cytokine expression in unstimulated samples, we found that mitogen-induced IL-2 responses by total CD8^+^ (*p* = 0.034) (Figure 3A) and naïve CD8^+^ T cells (*p* = 0.020) (Figure 3B) were significantly elevated among patients with CM-IRIS compared with controls, while there was a trend for higher CD8^+^ TDEM T cell IL-2 responses among subjects with CM-IRIS compared with controls (*p* = 0.061) (Figure 3C). These results suggest that T cells are primed to increase IL-2 expression during CM-IRIS. This could result either in expanding the number and function of GXM-specific T cell clones or could be an effect of IL-2 inhibiting T cell proliferation during CM-IRIS. 

### 3.4. Phenotype and Cytokine Responses among Subjects with Paired Samples at CM Diagnosis vs. CM-IRIS

We compared phenotype and cytokine expression at CM diagnosis and during CM-IRIS from 10 subjects with paired samples. CD4^+^ T cell frequency was significantly higher at CM-IRIS, 8% (IQR, 4–13%) compared to CM diagnosis, 3% (IQR, 1–3%) (*p* = 0.014). At CM-IRIS, the frequency of CD4^+^ T cells expressing HLA-DR was significantly decreased compared to CM diagnosis, 66% (IQR, 60%–79%) vs. 81% (IQR, 66%–90%), (*p* = 0.014) respectively. CD4^+^ T cells with a central memory phenotype expressing IL-17 were less frequent at CM-IRIS compared with CM diagnosis following GXM stimulation (Supplementary Figure S1). In contrast, without GXM stimulation, CD4^+^ T cells with effector phenotype expressing IL-2 were more frequent at CM-IRIS compared to CM diagnosis, 0.92% (IQR, 0%–2.4%) vs. 0%, (*p* = 0.016), respectively (Figure 3). No differences were observed in the frequency of CD4^+^ T_CM_ cells expressing IFN-γ on stimulation with GXM. 

### 3.5. CD4^+^ and CD8^+^ T Cell Polyfunctional Cytokine Responses at CM-IRIS

**Figure 4:**
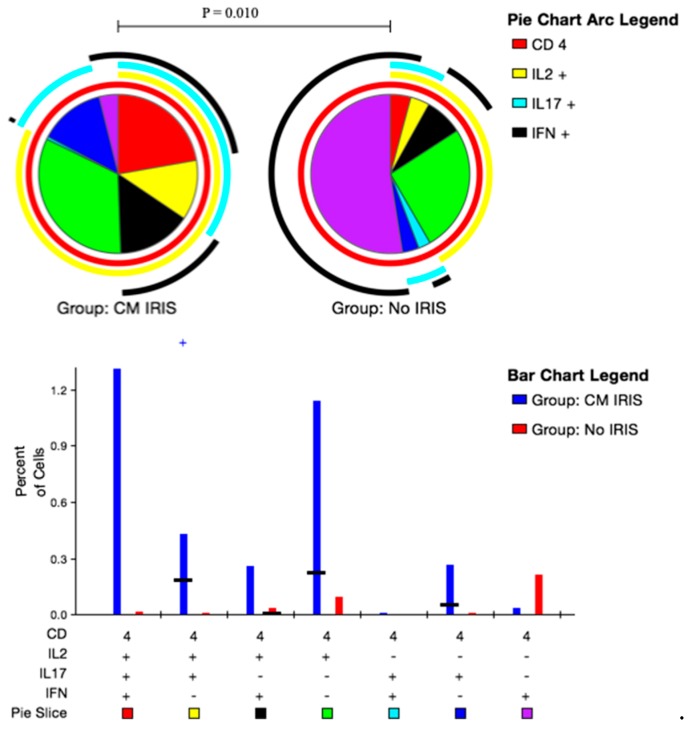
Peripheral blood mononuclear cells from subjects with cryptococcal meningitis were stimulated with Glucuronoxylomannan (GXM). Intracellular Interleukin-2 (IL-2), IL-17 and Interferon-γ (IFN-γ) expression by CD4^+^ T cells was quantified using flow cytometry. The bar chart shows each of the three possible combination responses on the x-axis. The percentage of the total cytokine response is shown on the y-axis, with the filled bar representing the interquartile range and a black line at the median. Statistically significant differences (*p* < 0.05) by rank-sum testing are indicated by the plus sign. Responses from ART matched control subjects who did not develop CM-IRIS are shown in blue, responses from subjects with CM-IRIS are in red on the bar graph. The pie charts show the fractions according to the pie-slice colors shown at the bottom of the bar chart, with color-coded arcs indicating the contributions of IL-2 (yellow), IFN-γ (black), and IL-17 (cyan) to the 3-, 2- and 1-function responses. Statistical comparisons of the overall responses by permutation testing are shown in the pie category test result chart where the red represents IL-2^+^IL-17^+^IFN-γ^+^ CD4^+^ T cells; yellow represents IL-2^+^IL-17^+^IFN-γ^−^ CD4^+^T cells; black represents IL-2^+^IL-17^−^IFN-γ^+^ CD4^+^ T cells; green represents IL-2^+^IL-17^−^IFN-γ^−^ CD4^+^ T cells; cyan represents IL-2^−^IL-17^+^IFN-γ^+^ CD4^+^ T cells; blue represents IL-2^−^IL-17^+^IFN-γ^−^ CD4^+^ T cells and purple represents IL-2^−^IL-17^−^IFN-γ^+^ CD4^+^ T cells. Patients with CM-IRIS had a significantly elevated proportion of duo functional IL-2^+^IL-17^+^IFN-γ^−^ CD4^+^T cells compared with matched Controls following GXM stimulation.

Upon stimulation with GXM, subjects at the time of IRIS CM-IRIS more frequently expressed dual-functional IL-2^+^IL-17^+^IFN-γ^−^ CD4^+^T cells vs. controls (0.34% vs. 0.02%; *p* = 0.010) (Figure 4). When we compared CD8^+^ T cell responses at CM-IRIS vs. controls, mono-functional IL-2^+^IL-17^−^IFN-γ^−^ CD8^+^ T cells were more frequent at CM-IRIS, 0.6% (IQR: 0.2, 1.0) compared to controls, 0.05% (IQR: 0.0, 0.1), *p* = 0.01 (Supplementary Figure S2). These data confirm the increased immune CD4^+^ T cell responses with co-expression of IL-2/IL-17 and CD8^+^ T cells expressing IL-2 during CM-IRIS thereby contributing to the immunopathogenesis of this syndrome. 

In summary, patients who developed CM-IRIS exhibited aberrant CD4^+^ T cell responses during the primary CM episode as demonstrated by their poor mitogenic and GXM-specific IL-2 responses. Notably, patients who developed CM-IRIS had diminished IFN-γ^+^ CD4^+^ T cells responsive to GXM at baseline and this response was markedly different at CM-IRIS. CM-IRIS was associated with robust dual-functional CD4^+^IL-2^+^IL-17^+^, CD8^+^IL-2^+^, and CD8^+^IL-17^+^ T cell responses. The selective expansion of these GXM-specific T cells is consistent with other studies of T cell responses during IRIS [13], which confirms exaggerated Th1 and Th17 responses during IRIS. 

## 4. Discussion

Cryptococcal IRIS remains a clinical challenge in populations where advanced HIV disease persists and yet the precise immunopathogenic mechanisms remain unclear. The phenotype of CD4^+^ T cell effector memory responses to *Cryptococcus* is associated with disease severity and outcome in HIV-associated cryptococcal meningitis [21]. We found that at baseline, those who subsequently developed CM-IRIS showed a characteristically lower immune response with decreased CD4^+^IFN-γ^+^ T cells and poor CD8^+^IL-17^+^ mitogenic responses at CM-IRIS. Indeed, during CM-IRIS, this pattern shifted to exhibit robust CD8^+^IL-2^+^ and CD4^+^IL-2^+^IL-17^+^IFN-γ^−^ T cell responses to GXM, which also distinguished them from the non-IRIS patients. We found no association between T cell memory phenotype and the incidence of CM-IRIS. 

Antigen-specific immune responses during CM-IRIS have been rarely studied. Most studies have looked at the expression of cytokines in blood or CSF but not the source of these cytokines. An instructive feature of this study was the detection of the cytokines expressed by T cells at the single cell level before and during cryptococcal IRIS.

To understand why some patients presenting with cryptococcal meningitis subsequently develop CM-IRIS after initiating ART, we evaluated the immune response to cryptococcal antigen. The high-molecular-mass capsular polysaccharide, glucuronoxylomannan, is found in high titers in patients with disseminated cryptococcosis [22,23]. The host response to GXM involves a granulomatous inflammatory response, intact cell-mediated immunity, and a Th1 pattern of cytokine release. GXM has anti-phagocytic properties and inhibits leukocyte migration and proliferation [24,25] and could explain the significantly diminished baseline immune response among patients who subsequently developed CM-IRIS. This diminished immune response could predispose to subsequent IRIS events resulting from failure to completely clear the antigen burden, which sets the stage for a possible over exaggerated response as hypothesized by Barber and colleagues when CD4 T cell recovery delivers the missing IFN-γ stimulus to partially activated macrophages that subsequently become fully activated *en masse* with a resulting ‘cytokine storm’ [26].

Consistent with this suppressive activity by GXM, participants who developed CM-IRIS could have had a high fungal burden at CM diagnosis (although not statistically significant) when compared to control subjects, suggesting that the presence of persistent antigen when immune reconstitution is initiated may underlie, in part, the development of IRIS. This finding is consistent with data showing that patients with disseminated cryptococcal fungemia have a six-fold higher risk of subsequently developing IRIS due to the high fungal burden and poor clearance. Similarly, patients with a cryptococcal antigen titer >1:1024 are reported to show an increased risk of IRIS [27]. These findings are also consistent with data showing that in the CSF of patients infected with *C. neoformans*, the pro-inflammatory cytokines IL-6, TNF-α, and IFN-γ were inversely correlated with cryptococcal fungal burden [28].

Murine models suggest that late cryptococcal clearance is impaired in the absence of IL-17 and, in a cohort of HIV-infected Ugandans, IL-17 was significantly lower in those who developed CM-IRIS compared with controls prior to ART initiation [29,30]. It is therefore plausible that more robust immune responses during the primary cryptococcal infection induced cryptococcal clearance and mitigated the risk of CM-IRIS among controls who had no IRIS.

It is also possible that patients who developed CM-IRIS had aberrant or dysregulated CD4^+^ T cell function during the primary cryptococcal infection resulting in persistent cryptococcal antigen, known to be a risk factor for CM-IRIS [27,31]. These findings are consistent with a study suggesting that robust CD4^+^ T cell responses during IRIS represent a dysregulated response against residual antigen [13]. This aberrant function appears to result in poor immune responses during the primary cryptococcal infection and subsequently in dysregulated robust T cell responses characteristic of IRIS. This T cell dysfunction could also explain the poor inflammatory response observed in the CSF of patients who developed CM-IRIS in a Ugandan cohort [15]. 

Interferon-γ plays an important role in the host defense against intracellular pathogens including *Cryptococcus neoformans* at the site of infection and has been studied as adjunctive therapy against cryptococcal meningitis [22,32]. At CM diagnosis, we found decreased frequencies of CD4^+^ IFN-γ^+^ T cells in patients who developed CM-IRIS compared with controls similar to the lower IFN-γ responses induced by cryptococcal mannoprotein in a cohort of patients with CM in Durban [14]. It should be noted that the cytokine responses in the Durban cohort were measured in whole blood cultures and not by intracellular flow cytometry at single cell level.

The differential gene expression between patients who develop CM-IRIS and those who do not has been demonstrated previously in a Ugandan cohort of HIV-infected patients. The most common molecular and cellular functions of the up-regulated genes were cell proliferation, cell apoptosis, and immune response (antigen presentation, innate responses, and inflammatory responses) [33]. More recent data from a Durban cohort suggests that CM-IRIS occurring within 12 weeks of ART initiation was predicted by the low expression of interferon-inducible genes, whereas late CM-IRIS events, occurring after 12 weeks of ART were characterized by abnormal upregulation of transcripts expressed in T, B, and natural killer cells such as IFNG, IL27, and LRB1 [34]. In addition to immunoactive cytokines, Yoon et al. have also demonstrated significantly low plasma levels of IgM, Lam-binding IgM, Lam-binding IgG, and GXM-IgG among patients who developed CM-IRIS [35]. Together, these data suggest the involvement of genetic, innate, and adaptive (T and B cell) mechanisms in the development of CM-IRIS.

Interleukin-2 has a dual role in the regulation of the immune system. On one hand, IL-2 is involved in the activation of the immune system by promoting the proliferation of lymphocytes, macrophages, and natural killer cells, as well as aiding in the differentiation of CD4 T cells [36]. On the other hand, IL-2 works to regulate the immune system through regulatory T cells, thus, inhibiting T cell proliferation. In the current study, GXM-induced CD4^+^ T cells expressing IL-2 were significantly elevated among participants at the time of CM-IRIS compared with controls. This immune response appeared to be driven by the naïve and terminally differentiated memory CD4^+^ T cells.

Similarly, we found significantly elevated CD4^+^ effector memory T cells expressing IL-17 among those with CM-IRIS compared with controls matched for ART duration. The differential expression of these cytokines in response to GXM suggests an exaggerated GXM-specific response among patients with CM-IRIS. This response could induce other pro-inflammatory cytokines that contribute to the immunopathology occurring at CM-IRIS. Indeed, a distinctive feature of CM-IRIS was the quality of the immune response at CM-IRIS compared to controls where we found significant elevation of dual-function CD4^+^IL-2^+^IL-17^+^ and monofunctional CD8^+^ IL-2^+^ T cells compared with controls without IRIS. This finding is consistent with data showing elevated IL-2 and IL-17 among subjects with CM-IRIS compared to time-matched controls in a cohort of HIV-infected Ugandans [30]. IL-2 and IL-17 have been implicated in promoting protective immune responses against *Cryptococcus* [29,37] in murine models. Thus, the differential expression of GXM-induced IL-2 and IL-17 among participants with CM-IRIS suggests that these two cytokines are involved in the immunopathogenesis of CM-IRIS. During CM-IRIS, IL-2 could play the role of inducing immune activation and driving the inflammatory process that is measurable in CSF. The Th17 differentiation factor, IL-6 is associated with IRIS [38,39,40] and inhibits T regulatory cell proliferation in favor of Th17 cell induction in the presence of TGF-β [41,42]. IL-17 is known to induce tissue inflammation and could also induce the inflammation observed during CM-IRIS [41]. IL-2 expression by CD8^+^ T cells was not elevated during TB-IRIS [43]. Although not specific to which cell type was expressing the cytokines, a study involving patients with pneumocystis, Histoplasma, *Mycobacterium avium*, and cryptococcal IRIS found similar elevation of IL-2 and IL-17 in plasma prior to and during the IRIS event compared to controls without IRIS [44], suggesting that our findings are not unique to cryptococcal IRIS.

The small sample size and low T cell counts preclude firm conclusions on differences between T cell phenotypes, markers of T cell activation and some of the cytokine responses among subjects with and without CM-IRIS. The ideal compartment to study would have been the CSF; however, due to the limited number of cells available in CSF and the challenge of obtaining CSF from ART-time matched controls, in vitro stimulation studies were performed with peripheral blood and are only reflective of the immune response in the periphery rather than the local immune response in the central nervous system, where CM-IRIS symptoms occur.

We only evaluated T cell responses against GXM. Approximately 90% of the cryptococcal cell wall is comprised of GXM [45]. Thus, the T cell responses in this study may not be reflective of what occurs in vivo where responses against other cryptococcal capsular antigens including galactoxylomannan and mannoproteins may be dissimilar. It is also possible that the cellular and cytokine responses we observed with CM-IRIS may represent a more natural or healthy response to this pathogen as the subjects’ immune function was restored following ART. We did not evaluate T regulatory cells, which would have given a broader picture of the adaptive immune response and the balance between pro-inflammatory and anti-inflammatory pathways, which are now thought to contribute to fungal IRIS [46]. Larger prospective studies are needed to concurrently examine T cell responses against various cryptococcal antigens in both CSF and peripheral blood prior to ART initiation, after patients initiate ART and at CM-IRIS events.

In conclusion, cryptococcal IRIS after ART initiation occurs as a consequence of both host and pathogen factors. Aberrant T cell function during the primary cryptococcal infection observed in our study could have contributed to the pathogenesis of CM-IRIS. The elevated expression of GXM-specific IL-2 during CM-IRIS may indicate GXM-specific T cell proliferation during the IRIS event. These responses occurring in the enclosed CNS may be detrimental, resulting in the subsequent immunopathology associated with cryptococcal IRIS.

## Figures and Tables

**Table 1:** Characteristics of subjects who developed cryptococcal meningitis (CM)-immune reconstitution inflammatory syndrome (IRIS) vs. subjects without CM-IRIS.

	Controls (*n* = 6)	CM-IRIS (*n* = 11)	*p*-Value
Men, N (%)	1 (17%)	8 (73%)	0.05
Age, years	35 (28, 40)	35 (29, 42)	0.937
CD4^+^ T cells/μL-Diagnosis	8 (5, 166)	6.5 (4, 28)	0.828
- >3 month on ART	156 (55, 309)	68 (33, 79)	0.256
CD8^+^ T cells/μL-Diagnosis	163 (97, 784)	256 (140, 591)	0.515
- >3 month on ART	1005 (615, 1086)	831 (565, 997)	0.463
Plasma HIV RNA (log_10_ copies/mL)	5.1 (4.6, 5.2)	5.3 (4.8, 5.6)	0.260
CSF Cryptococcus (log_10_ CFU/mL)	3.97 (2.45, 5.26)	5.33 (4.96, 5.46)	0.078
CSF CRAG titer, 1:x	4512 (528, 12192)	7200 (4048, 16384)	0.455
CSF protein (mg/dL)	60 (47, 68)	53 (20, 70)	0.471
CSF WBC/μL	20 (<5, 45)	<5 (<5, <5)	0.169
Duration from ART initiation (days)	67 (48, 92)	78 (43, 202)	0.737

Values listed as median (IQR) or mean (±SD). Values are at the time of cryptococcal meningitis diagnosis unless otherwise stated. Abbreviations: ART-Antiretroviral Therapy; CRAG-Cryptococcal Antigen; CSF-Cerebrospinal Fluid; CFU-colony forming units; HIV-Human immunodeficiency virus.

**Table 2:** Cytokine responses by T cell phenotype among subjects with CM-IRIS vs. controls following GXM stimulation at CM diagnosis.

	Controls (*n* = 5)	CM-IRIS (*n* = 10)	*p*-Value
**CD4^+^ T cells**			
**Central memory (T_CM_)**			
IFN-γ^+^	6 (3, 11)	0.8 (0, 3)	0.005
IL-2^+^	5 (2, 22)	1 (0.1, 3)	0.012
IL-17^+^	2 (1, 6)	0.5 (0, 2)	0.054
**Effector memory (T_EM_)**			
IFN-γ^+^	8 (4, 16)	0.5 (0, 3)	0.027
IL-2^+^	3 (2, 13)	0 (0, 0.1)	0.004
IL-17^+^	2 (1.9, 3)	0	<0.001
**Terminally differentiated effector memory (T_TDEM_)**			
IFN-γ^+^	3 (1, 16)	0 (0, 0.4)	0.005
IL-2^+^	74 (12, 86)	0	<0.001
IL-17^+^	0.1 (0, 1.1)	0 (0, 2)	0.624
**CD8^+^ T cells**			
**Central memory (T_CM_)**			
IFN-γ^+^	2.4 (2.2, 3.1)	0.4 (0.1, 0.5)	<0.001
IL-2^+^	1.1 (0.5, 2.1)	0.07 (0.03, 0.3)	0.005
IL-17^+^	1.2 (0.9, 1.4)	0.08 (0.02, 0.2)	0.003
**Effector memory (T_EM_)**			
IFN-γ^+^	6.2 (3.1, 9.9)	0.7 (0.2, 1.5)	0.005
IL-2^+^	1.2 (0.5, 1.7)	0.01 (0, 0.1)	<0.001
IL-17^+^	1.2 (1.0, 1.3)	0.06 (0, 0.2)	<0.001
**Terminally differentiated effector memory (T_TDEM_)**			
IFN-γ^+^	1.3 (0.6, 3.5)	0.3 (0.2, 0.9)	0.037
IL-2^+^	1.1 (0.3, 1.2)	0.03 (0, 0.6)	0.068
IL-17^+^	0.09 (0.08, 0.25)	0.01 (0, 0.17)	0.119

Figures are presented as percentages of T cells. *P*-values obtained by Mann Whitney *U* test. Data are presented as median (Interquartile Range). Significant *P* values are in bold typeface.

## Supplementary Materials

The following are available online at https://www.mdpi.com/2309-608X/5/2/42/s1, Supplementary Figure S1: Frequency of CD4^+^ IL-17^+^Central memory and CD4^+^ IL-2^+^ Effector memory cells from paired samples at CM Diagnosis vs. CM-IRIS. Supplementary Figure S2: CD8^+^ T cell Cytokine responses at CM-IRIS vs. Controls in response to GXM Stimulation.
