# Supplementary figures and images for: HIV-Associated Cryptococcal Immune Reconstitution Inflammatory Syndrome Is Associated with Aberrant T Cell Function and Increased Cytokine Responses
Source: J Fungi (Basel). 2019 May 23;5(2):42. doi: 10.3390/jof5020042 (PMC6616503; doi:10.3390/jof5020042)

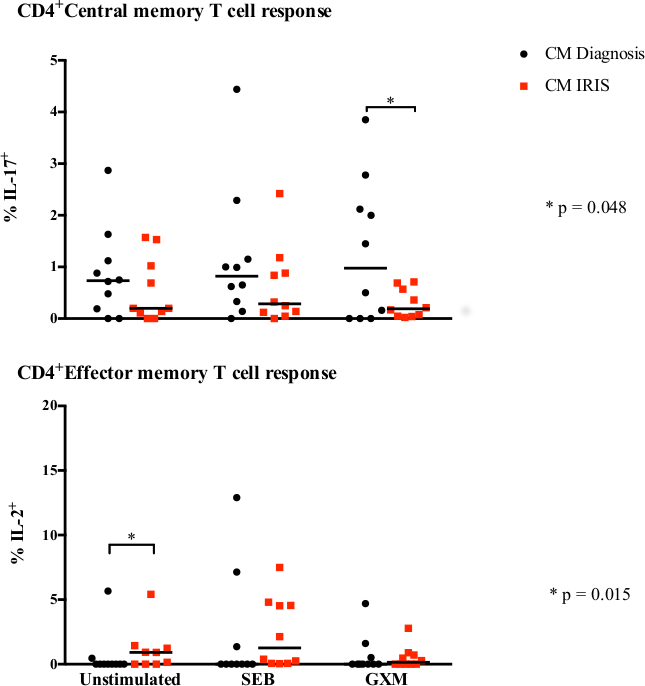

Supplement: Supplementary file 1 [file jof-05-00042-s001.zip › Supplementary Figure S1.tiff]

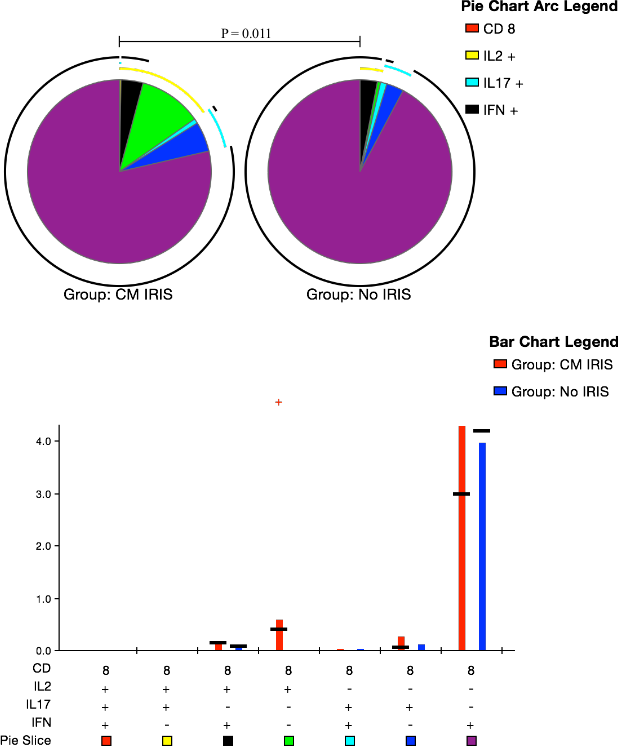

Supplement: Supplementary file 1 [file jof-05-00042-s001.zip › Suppmentary Figure S2.tiff]
